# Cytotoxicity of Extracts from New Zealand Surf Clams Against Organ Cancer Cell Lines

**DOI:** 10.3390/biomedicines7020025

**Published:** 2019-03-30

**Authors:** Tinu Odeleye, William Lindsey White, Jun Lu

**Affiliations:** 1 School of Science, Faculty of Health and Environmental Sciences, Auckland University of Technology, Auckland 1010, New Zealand; tinu.odeleye@aut.ac.nz (T.O.); lindsey.white@aut.ac.nz (W.L.W.); 2 College of Life Sciences and Oceanography, Shenzhen University, Shenzhen 518071, China; 3 School of Interprofessional Health Studies, Faculty of Health and Environmental Sciences, Auckland University of Technology, Auckland 1010, New Zealand; 4 Institute of Biomedical Technology, Auckland University of Technology, Auckland 1010, New Zealand; 5 College of Food Engineering and Nutritional Science, Shaanxi Normal University, Xi’an 710119, China

**Keywords:** extract, bioactive, surf clam, organ cancer cells, nutraceutical, New Zealand

## Abstract

In this study, we examined the cytotoxic effects of four fractions from three species of New Zealand (NZ) surf clam on four common organ cancer cells. In most cases, a dose- and time-dependent inhibition on the proliferation of the cancer cells was observed. This was most significant in WiDr (colon) cells, where the percentages of viability reduced to as low as 6%, 5%, and 17% (at 1000 µg 72 h) by extracts from Diamond shell, Storm shell, and Tua tua species, respectively. A549 (lung) cells were the least susceptible to the treatment, with viability percentages at 82%, 15%, and 45%, under the same conditions. Induction of caspase-dependent apoptosis and alterations to the cell cycle further supported the observed morphological analysis. The ethanol, petroleum ether, and ethyl acetate fractions of NZ surf clam, rich in lipids and proteins, were more potent than their water-based counterpart. This is the first demonstration where extracts from NZ surf clams show the ability to inhibit the growth and proliferation of cancer cell lines. We suggest that NZ surf clam extracts have the potential to be further studied and developed as candidates for cancer supplementary management/treatment.

## 1. Introduction

Marine animals possess bioactive substances (such as oligosaccharides, vitamins, and fatty acids) capable of reducing the risks of certain diseases [1]. These bioactives are diverse in structure, chemical and biological compositions, and possess extremely potent bioactive capabilities. Immunomodulatory, anti-viral [2], anticoagulant [3,4], antithrombotic [5], antihypertensive [6], antibacterial [7], anti-inflammatory [8], antidiabetic [9], and antioxidant [10] activities have been reported.

The extraction (primitive or otherwise) and/or use of bioactive substances from marine organisms is a practice dating back to ancient Egyptian [11], Crete [12], Indian [12], and Chinese times [13]. Mollusks rank high on the list of marine organisms explored for their bioactive metabolites. This may be because mollusks are more likely to produce metabolites to protect themselves from predation and pathogens [13]. Clams, among other mollusks, have been exploited for their bioactive properties [14,15,16,17,18,19]. There are seven main species of surf clams in New Zealand (NZ), and the NZ surf clam market is growing globally [10]. NZ’s most harvested and exported surf clams, the Diamond shell (*Crassula aequilatera*), Storm shell (*Mactra murchisoni*), and Tua tua (*Paphies donacina*), are used in this research.

Gastrointestinal (GI) cancer is a term describing a group of cancers that affect the digestive system. This includes the gall bladder, esophagus, liver, pancreas, stomach, small intestine, colon, and rectum [20]. Cancers of the GI tract are the most common form of cancer in men and women alike [20], and are a significant cause of mortality and morbidity globally [21]. GI cancers account for roughly 15% of diagnosed cancer in men and women [22]. Lung cancer includes small cell lung cancer and non-small cell lung cancer. The estimated deaths of lung cancer in 2018 were 154,050, constituting 25.3% of all cancer deaths [23]. Surf clam extracts’ anticancer activities have been described in the literature [24,25,26]. Our previous studies indicate that NZ surf clam extracts possess antioxidant activities [10]. This study aims to, for the first time, determine and evaluate the effects of extracts from three NZ surf clam species on four types of cancer cell lines, including three GI/digestive cancer cell lines (Liver- Hep G2, Pancreatic- MIA PaCa-2, and Colon- WiDr) and one respiratory cancer cell line (Lung- A549) in vitro.

## 2. Results and Discussion

### 2.1. Antiproliferative Activities of NZ Surf Clam Extracts

**Figure 1:**
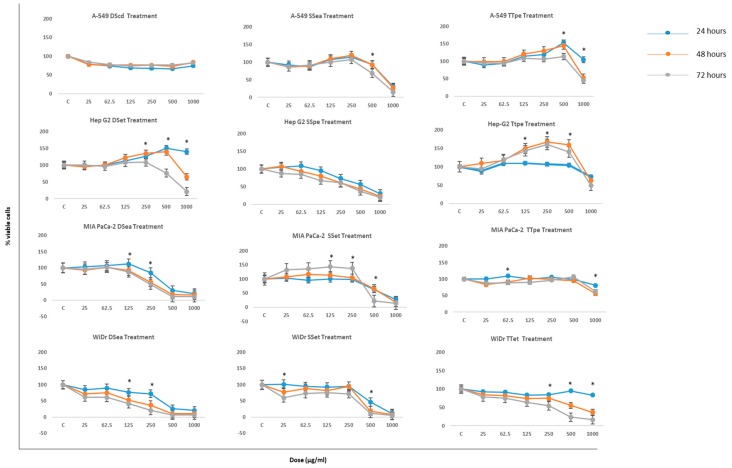
The inhibitory effect of New Zealand (NZ) surf clam extracts on the growth of A-549, Hep G2, MIA PaCa-2, and WiDr after an incubation time of 24, 48, and 72 h. Cells were incubated in the presence of various extract concentrations. A relative cell viability of 100% was designated as the total number of cells that grew after each time point. Each experiment was carried out twice, in triplicate. Data is presented as means ± SE. * indicates statistical significance, *p* < 0.05.

Initial examinations of the effects of NZ surf clam extracts on the proliferation of the selected cancer cells were carried out employing the 3-(4,5-dimethylthiazol-2-yl)-2,5-diphenyltetrazolium bromide (MTT) assay. The cell lines were treated with 12 extracts each (Supplementary Figure S1a–d), but only the best three extracts are discussed for brevity. Most extracts displayed a dose- and time-dependent inhibition on the proliferation of the cancer cells used in this study (Figure 1). Results are expressed as percentages of viable cells compared to the control.

In order to ensure an accurate context for cellular viability in this study, control treatments of cells were carried out using only the extracting solvents. MTT assays were performed, and determined that solvent-treated cells did not induce apoptosis (results not shown). Additionally, extract only (no cells present) did not chemically reduce MTT (results not shown). This is to ensure that the observed activities are a function of their constituent compounds.

Effects of NZ surf clam extract treatment, extracted using ethanol (et) petroleum ether (pe), and ethyl acetate (ea), on A-549, Hep G2, MIA PaCa-2, and WiDr suggest significant inhibition. Cell lines were treated with a series of concentrations between 25 and 1000 µg/mL, and assessed employing the MTT assay after 24, 48, and 72 h of treatment.

The MTT differential sensitivities, based on a 72-h time point, were least to greatest as follows: A549 > Hep G2 > MIA PaCa-2 > WiDr. MIA PaCa-2, and WiDr cell lines were the most susceptible to treatment with NZ surf clam extracts, showing dose-dependent inhibitions after only 24 h of treatment. Inhibition values of DSea-treated MIA PaCa-2 and WiDr at 250, 500, 1000 µg/mL (72 h) were 51%, 89%, 89%, and 80%, 94%, 94%. SSet-treated MIA PaCa-2 and WiDr cells also showed incredible inhibition values of 73%, 83%, 86%, and 89%, 93%, 95%, respectively at 1000 µg (24, 48, and 72 h). After only 48 h of treatment at 250 µg/mL, TTet- treated WiDr cell proliferation was inhibited by 63%. The best inhibition rates at 24, 48, and 72 h (1000 µg/mL) was observed in SSet-treated WiDr in all cases. In MIA PaCa-2, the inhibition rate of DSea- treated cells, was marginally better than its SSet counterpart. Similar results of high cell viability inhibition were obtained by hard clam (*Meretrix lusoria*) extract-treated AGS and HL-60 cells at similar concentrations [26].

A549 and Hep G2 were the least susceptible cell lines to treatment. At 1000 µg/mL (72 h), of their respective Diamond Shell (DS), Storm Shell (SS), and Tua Tua (TT) treatments, both cell lines displayed percent viabilities of 82%, 15%, 45%, and 31%, 19%, and 49%. These inhibition values were already achieved by MIAPaCa-2 and WiDr treated cells at 24 h. SSpe-treated Hep G2, on the other hand, showed remarkable cell viability inhibition throughout the course of study, showing percentage inhibition values of 73%, 57%, 31% (24 h), 61%, 44%, 23% (48 h), and 61%, 38%, 19% (72 h) at 250, 500, and 1000 µg/mL, respectively. A549 was even less sensitive to treatments than Hep G2, with the its best inhibitory effect at 71%, 74%, and 85% after 24%, 48%, and 72 h of treatment, respectively.

Clam extract-treated MCF-7 and MBA-MB-231 at 1500 µg/mL, cell proliferation inhibition values were 78.8% and 64.3%, respectively (72 h treatment) [25]. Such values were obtained in this study at 1000 µg/mL, after only 24 h of treatment in SSea-treated A549, SSpe-treated Hep G2, DSea-treated MIA PaCa-2 and WiDr, and SSet-treated WiDr cells. Moreover, compared to BCP-A (Blood clam- *Tegillarca granosa*) -treated (5 mg/mL) H1299, HeLa, and DS-145 cells, which showed inhibition rates of 77.28%, 90.58%, and 87.54%, respectively [27], the NZ surf clam extracts performed significantly better, showing similar inhibition values at 500 and 1000 µg/mL (in MIA Paca-2 and WiDr cells) after 24 h of treatment in both cases.

In most cases, no obvious dose-dependent inhibition was observed at concentrations up to 250 µg/mL (Figure 1). In all cases, the inhibitory effect was most significant in concentrations above 125 µg/mL. Overall, concentrations between 25 and 125 µg/mL promoted the growth of cancer cell lines. Similar observations were made by Liao et al. [25], where breast cancer cells were unaffected by *Corbicula fluminea* extract treatments at concentrations ≤250 µg/mL. This temporary increase in cells’ survival may indicate that the cells go through a short-term healing or recovery before death [28], or that lower concentrations are simply ineffective, thereby promoting cell growth, and resulting in treatment resistance in the cells. Another possible reason might be that at lower concentrations, the cells activate pro-survival pathways, which are shut down at higher concentrations.

The cytotoxicity of *Cratoxy formosum* extract against Hep G2 cells at 500 µg/mL, after 48 h of treatment is similar to those observed in this study (SSpe-treated Hep G2) at same concentration and time [29] Similar cytotoxicity was observed at 250 µg/mL in DSea-treated WiDr cells at 48 h.

The results indicate that DSea and SSet fractions exhibited great suppression on cell viability especially in the MIA PaCa-2 and WiDr cell lines at 250, 500, and especially 1000 µg/mL (at all time points tested) (Figure 1). The results also show that NZ surf clam extracts exhibited a broad spectrum of cytotoxicity against different cancer cells under identical conditions.

### 2.2. Morphological Analysis

**Figure 2:**
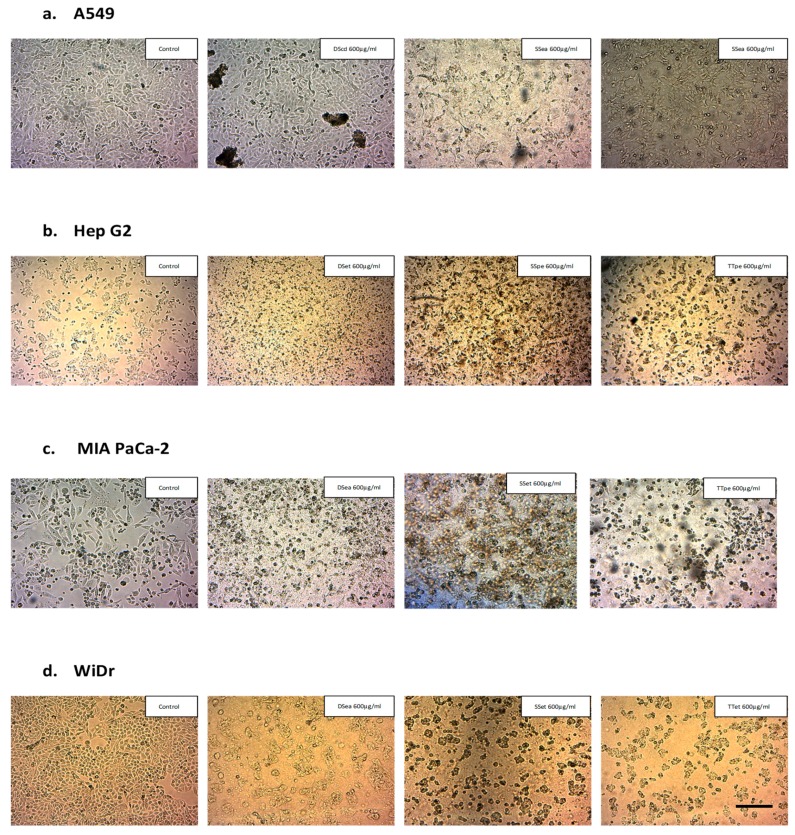
Morphological examination of extract-treated cancer cell lines, A549 (**a**), Hep G2 (**b**), MIA PaCa-2 (**c**), and WiDr cells (**d**) (scale bar: 20 µm)

Chromatin condensation, nuclear compaction and fragmentation, formation of blebs, shrinkage of cytoplasm, and growth decline are all morphological hallmarks of apoptosis [30]. The morphological examination of NZ surf clam-treated cancer cell lines was carried out to observe signs of apoptosis. Cells were treated with respective extracts and incubated for 72 h. The cell morphology was observed using an inverted microscope at 40× magnification (Zeiss). It was observed that untreated controls appeared morphologically normal, while treated cells showed signs of apoptosis, such as reduction in cell number, cell shrinkage and disintegration, and severe loss of adhesion (Figure 2).

### 2.3. Apoptosis Inducing Activity of NZ Surf Clam Extracts

Apoptosis is the most common anticancer mechanism in many cancer therapies [31]. To confirm that exposure to NZ surf clam extracts induced the death of A549, Hep G2, MIA PaCa-2, and WiDr cells by apoptosis in vitro, an apoptosis assay was performed. Cells were treated with two concentrations of their respective DS, SS, and TT extracts (400 and 600 µg/mL), and then stained with Annexin V and PI. Annexin V identifies apoptotic cells by binding to phosphatidyl serine exposed on the outer leaflet of the plasma membrane. PI stains dead cells by binding to the nucleic acids in the cell [32].

**Figure 3:**
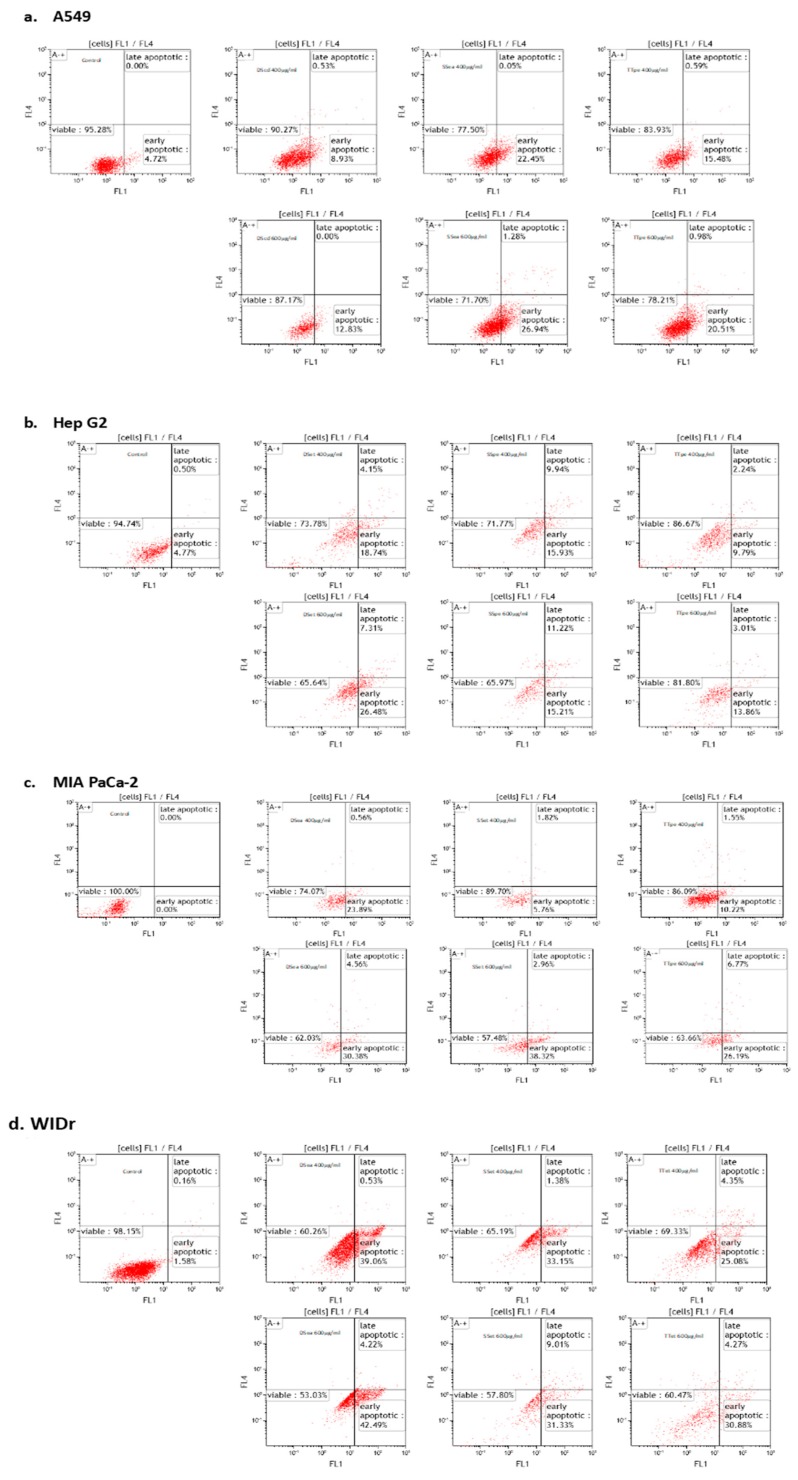
Induction of early apoptosis in A549, Hep G2, MIA PaCa-2, and WiDr cells by NZ surf clam extracts. Annexin V/Dead Cell Apoptosis Kit with Aleza^®^ Fluor 488 annexin V and PI determined the percentage of early apoptotic cells, examined by flow cytometry. Each experiment was carried out twice, in duplicate. Data are represented as mean ± SE.

The percentage of apoptosis was examined after 7 h of treatment, based on earlier optimization studies (data not shown). As shown in Figure 3, cell distribution was altered, showing a decrease in viability. Notably, there was a very low percentage of late apoptotic cells in all cell lines tested. This may be due to a relatively short treatment time of 7 h, correlating the early stages of apoptosis in the cell lines tested. Given the nature of this assay, it is plausible that a longer treatment time would reflect higher populations of late apoptotic cells (Figure 3). Therefore, only two populations are discussed in this study: Viable and early apoptotic cells. Furthermore, cells in this study did not undergo preliminary serum starvation; therefore, the apoptosis observed was initiated by the treatments, rather than in association with cell culture conditions.

A549 cells were the least responsive to treatments, corresponding with the MTT data. The viability of A549 cells did not indicate apoptosis to the degree in which was seen in the other cell lines. The cell viability percentages in A549 cells after a 400 µg/mL treatment were 90.27% (DScd), 77.50% (SSea), and 83.93% (TTpe). WiDr, the most susceptible cell line, on the other hand, showed percentage viability of 60.26% (DSea), 65.19% (SSet), and 69.33% (TTet) under the same conditions. The apoptosis observed in DS, SS, and TT-treated MIA PaCa-2 cells at 600 µg/mL (30.38%, 38.32%, and 26.19%) was similar to WiDr treated cells (42.49%, 31.33%, and 30.88%). In like manner, Hep G2 demonstrated high sensitivities to extracts with viable cells of 65.64% (DSet), 65.97% (SSpe), and 81.80% (TTpe) at 600 µg/mL. This may suggest that slightly different mechanisms are associated with different cell lines. Notably, A549 is a lung cancer cell line, while the others are GI-associated, and likely share more commonality in response cascades. These differences appear to be associated with caspase-3/7 cascade activation in the different cell lines (as is discussed in Section 2.4).

NZ surf clam extracts produced great apoptosis results after the optimized 7 h of treatment, compared to other studies. Protein hydrolysates from the blood clam (*Tegillarca granosa*), for example, achieved 14.29%, 17.71%, and 20.28% early apoptotic cells at 1.5, 2, and 2.5 mg/mL, after 20 h of treatment [33]. Moreover, the percentages of early apoptotic cells in Sepia ink oligopeptide-treated DU-145, PC-3, and LNCaP at 15 mg/mL were 38.26%, 39.96%, and 16.11%, respectively [27]. Similar and greater percentages of early apoptotic cells were achieved in this study at 400 and 600 µg. The induction of apoptosis after only several hours suggests that apoptosis was induced by pre-existing apoptosis machinery [34].

### 2.4. Caspase-3/7 Activity of NZ Surf Clam Extracts

To further confirm apoptotic activities by NZ surf clam extracts, we assayed the caspase-3/7 activity employing the Apo-ONE Homogeneous Caspase-3/7 Assay kit. The level of caspase-3/7 activity was estimated after 24 h of treatment for each cell line. The results suggest that NZ surf clam extracts induced apoptosis in a caspase-dependent manner.

In this study, the caspase-3/7 activities in all four cell lines correspond closely with the apoptosis results. The major difference in caspase-3/7 activities was seen between A549 and MIA PaCa-2 cells. TTpe- treated A549 at 400 and 600 µg/mL, showed higher caspase activities (344.7% and 375.3%) compared to TTpe-treated MIA PaCa-2 treated cells (216.1% and 147.9%). The caspase activities of DS- and SS- treated MIA PaCa-2, however, exceeded those of the A549 cells. As expected, WiDr cells possessed the highest caspase-3/7 activities in this study.

The most obvious increase in caspase activity was observed in DSea (864.5% and 1268.3%) and SSet (1435.8% and 973.2%)-treated WiDr cells, at both 400 and 600 µg compared to the control (100%). The extract-induced apoptosis in the cell lines studied herein seem to be related to the mitochondria-mediated pathway, based on the activation of caspases-3 and -7.

**Figure 4:**
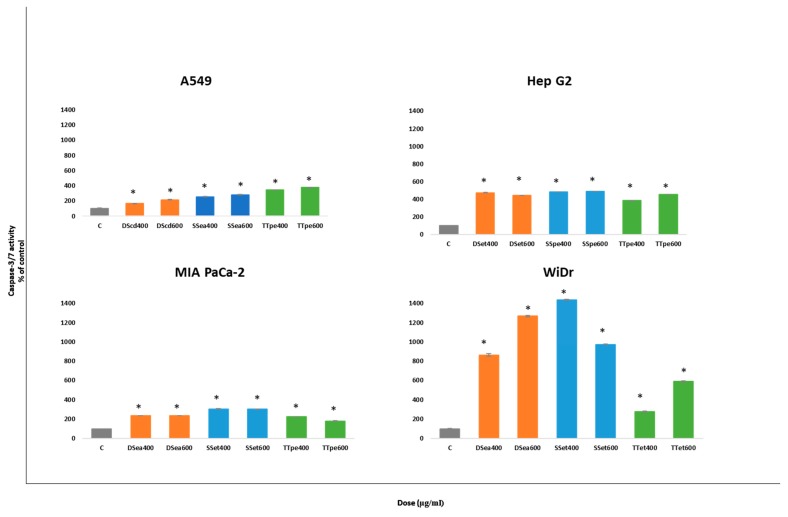
Caspase 3/7 activities in A549, Hep G2, MIA PaCa-2, and WiDr cells after treatment with NZ surf clam extracts. Cell lines were treated with two concentrations (400 and 600 µg for 24 h. Caspase 3/7 activities were evaluated by Apo-ONE Homogeneous Caspase-3/7 Assay kit. The caspase-3/7 activity of each group was indicated by their rate fluorescence (RFU). Each experiment was carried out twice, in triplicate. Data is presented as mean ± SE. * indicates significant difference to control groups, *p* < 0.05.

In this study, A549 cells showed lower caspase-3/7 activities (Figure 4a). However, these data would be indicative of the activity, and not the expression of the caspase-3/7 proteins, isolating and defining A549 cells, relative to the expressions defining the GI cancers tested. Albeit, A549 displayed relatively lower inhibitions and apoptotic activities (Figure 1 and Figure 3), its apoptotic activity was caspase-dependent and concentration dependent. These findings suggest greater activation of caspase-3/7 cascades in GI associated cancer cell lines by the extracts, in preference to lower activations in lung cancer cells.

Compared to the caspase-3/7 activities of *Angelica dahurica* extracts on colon cancer cells (HT-29) (48 h treatment), the NZ surf clam extracts performed significantly better, showing approximately 3, 5, and 1-fold increases in caspase-3/7 activities at the same concentration (400 µg/mL) in DSea, SSet, and TTet- treated WiDr (colon cancer) cells (24 h treatment) [35]. Furthermore, comparison of *Cratoxy formosum*-treated Hep G2 to NZ surf clam-treated Hep G2, revealed interesting results. Buranrat et al. [35] demonstrate a 2.9-fold increase in caspase activity over their control at 100 µg treatment. Our studies highlight 400 µg/mL treatments of Hep G2 cells resulted in an approximate 4-fold increase in caspase activities, across DSet, SSpe, and TTpe extracts. Although our observed activities occured at a higher concentration, there is an indication of potential similarities or improvement over the *Cratoxy formosum* extract.

### 2.5. Cell Cycle Alterations by NZ Surf Clam Extracts

**Figure 5:**
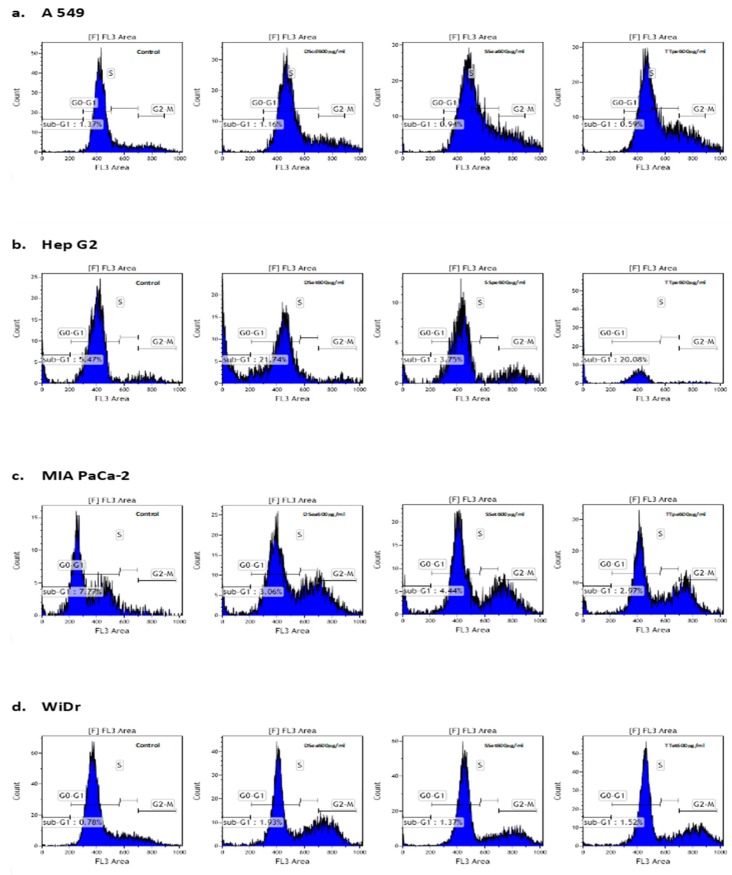
Induction of cell cycle arrest on (**a**) A549 cells (DScd, SSea, and TTpe treatments); (**b**) Hep G2 cells (DSet, SSpe, and TTpe treatments); (**c**) MIA PaCa-2 cells (DSea, SSea, and TTpe treatments), and (**d**) WiDr cells (DSea, SSet, and TTet treatments), respectively by NZ surf clam extracts. Cell lines were treated with 600 µg/mL of various extracts. The percentages of cells in the different phases of cell cycle was determined by PI staining and examined by flow cytometry. Each experiment was carried out twice, in duplicate. Data are represented as mean ± SE.

The inhibition of cell growth and proliferation is significantly characterized by interference to the normal cell cycle [36]. In this study, the cell distribution was analyzed after a 72-h treatment. The percentage of cells in their different phases indicates an alteration to normal cell population distribution (Figure 5).

The cell cycle assay helps in the analysis of cell cycle parameters of surviving cells, the capacity to inhibit the progression of the cell cycle, and the proportions of sub-G1 (indicative of cell death) cells. The sub-G1 population is generally used as an index of apoptotic DNA fragmentation [25]. There was little to no increase in the sub-G1 cells in all cases, but DSet- and TTpe-treated Hep G2 cells (600 µg/mL) (Figure 5b). The sub-G1 cell populations in both cases were 21.7% and 20%, respectively. In all cases, including Hep G2 treated cells, there was a significant decrease of cells in the G0-G1 population, indicating cell proliferation inhibition. In MIA PaCa-2 cells, for example, there was a 1.4 (DSea treatment), 1.4 (SSet treatment), and a 1.5 (TTpe treatment) fold decrease in the G0-G1 population, compared to the control. These results also reveal that NZ surf clam treatment induced the accumulation of cells in the G2-M and S-phases, except in DSet- and TTpe-treated Hep G2 cells. In the SS treatment of all cell lines, there was a 163.29% (A549), 207.99% (Hep G2), 544.50% (MIA PaCa-2), and 285.81% (WiDr) increase of cells in the G2-M phase compared to their respective controls. The accumulation of cells in the G2-M phase suggests that the extract-exposed cells are not re-entering the G1-G0 phase of the cell cycle. The accumulation of cells in the G2-M- and S- phases, and ergo the strong G2/M and S- phases cell cycle arrest, may be the major mechanism through which the proliferation of A549, MIA PaCa-2, and WiDr cancer cells is inhibited. This accumulation of cells in the G2-M and S-phases correlate well with findings by Huang et al. [27] (Sepia ink-treated DU-145 and LNCaP), and Liao et al. [25] (*Corbicula fluminea* extracts -treated MCF-7).

### 2.6. Chemical Composition of Extracts

The fractions obtained from the entire extraction process were previously characterized [10]. The crude extract obtained from DS, SS, and TT, contained comparable amounts of carbohydrates (15.48%, 15.80% and 17.10%) and proteins (16.25&, 15.48%, 16.48%). Similar protein content was found in the DSea, SSea, and TTea fractions (12.49%, 15.58%, and 17.89%). The major content of the et fraction was lipid, with most of it extracted in the pe fraction (84.19%- DSpe, 63.50%- SSpe, and 72.01%- TTpe). A significant amount of lipids was also found in the ea fractions (21%- DSea, 19.50%- SSea, and 56.49%- TTea).

In this study, the et, pe, and ea fractions exhibited the most significant growth inhibitions across all four cell lines. The ea fraction was particularly comprised of proteins and lipids, suggesting that either the protein- or the lipid-, or both components were responsible for the cytotoxicity observed. Other studies show that the lipid extracts of molluscan extracts possess fat, sterols, sterol esters, and steroids, responsible for the observed bioactivity [37,38,39,40]. The pe fraction clearly contains the most lipids than any other fraction. It is highly plausible that lipids are responsible for some bioactive component which elicits the observed responses. Notably, the TTpe fraction was the most effective TT fraction in A549, Hep G2, and MIA PaCa-2. TTpe-treated WiDr showed higher susceptibility at 1000 µg/mL than TTet-treated WiDr at 72 h (results not shown). However, cumulatively, TTet-treated WiDr was chosen because of its remarkable effects over a range of concentrations rather than effectiveness over a single higher concentration. This is more desirable as we have activities at lower concentrations in TTet-treated WiDr over its TTpe-treated WiDr counterpart.

## 3. Materials and Methods

### 3.1. Materials

A549, human lung cancer cells (Cat No. CRL-185); WiDr, human colon cancer cells, (Cat No. CCL-218); Hep G2, human liver cancer cells (HB-8065); and MIA PaCa-2, human pancreatic cancer cells (CRL-1420) were purchased from American type culture collection (ATCC) (Manassas, VA, USA). Ribonuclease A from bovine pancreas (Cat No. R4875- 100mg) and Triton™ X-100 for molecular biology (Cat No. T8787-250ML) were purchased from Sigma-Aldrich (St Louis, MO, USA). MTT (3-(4, 5-Dimethylthiazol-2-yl)-2, 5-Diphenyltetrazolium Bromide) Formazan powder (Sigma Aldrich, St. Louis, USA); Roswell Park Memorial Institute (RPMI) 1640 medium, no phenol red (Life technologies, Auckland, NZ); Petroleum ether and Ethyl acetate (Global Science, Auckland, New Zealand), ethanol (ThermoFisher, Auckland, New Zealand). Foetal Bovine Serum (FBS) (Medica Pacifica, Auckland, New Zealand), l- Glutamine (200 mM; 100 mL) (Life technologies, Auckland, New Zealand), Penicillin- Streptomycin (10,000 U/mL; 100 mL) (Life technologies); TrypLE™ Express, no phenol red (Life technologies), Dimethyl sulfoxide (DMSO) (Merck chemicals); Trypan blue stain (0.4%) (Life technologies); Dulbecco’s Phosphate Buffered Saline (D-PBS) (Life technologies), Apo-ONE^®^ Homogeneous Caspase-3/7 Assay kit (In vitro technologies, Auckland, New Zealand), Alexa Fluor^®^ 488 Annexin V/ Dead Cell Apoptosis kit (Thermo-Fisher Scientific).

### 3.2. Extraction and Fractionation of Clam Samples

Three New Zealand surf clam species provided all samples characterized in this study, including Storm shell (SS) (*Mactra murchisoni*), Diamond shell (DS) (*Crassula aequilatera*), and Tuatua (TT) (*Paphies donacina*). Blanched clam meat was deshelled and drained of excess fluids. After allowed to come to room temperature, the flesh was oven dried in a hot air oven at 60 °C to a constant weight. Flesh was pulverized. Milled clam powder was stored at -−20 °C until use. All clam extraction and measurements were carried out in dim light to reduce any possibility of oxidation. The extraction method from a previous study was adopted with slight modifications [41]. Initial extraction fractions were generated in parallel using water (cd) and ethanol (et) as solvents. Clam powder solubilized in distilled water was stirred constantly at room temperature using a magnetic stir-bar for one hour. The supernatant was removed, replaced with fresh solvent, and stirred for another hour. This process was repeated until the solvent was colourless. The supernatant was collected by centrifugation. Ethanol extraction was carried out in the same way. The clear solution was collected and evaporated under reduced pressure using a Rota evaporator (Buchi Rotavapor R-215, Global Science, Auckland, New Zealand) until complete dryness. The water (cd) and ethanol extracts (et) were collected after evaporation and stored at −20 °C. The ethanol extraction (et) of each clam species were further fractionated by liquid–liquid extraction steps. The ethanol extract was further fractioned according to the polarities of petroleum ether (pe) and ethyl acetate (ea). The extracts were dissolved in 100mL distilled water and fractionated with petroleum ether. Extracts (‘pe’) were collected and concentrated under reduced pressure. Further step-by-step fractionation was done using ethyl acetate, which resulted in the fractions of ‘ea’. Each fractionation process was repeated until the solvent was colourless. Fractions were evaporated to dryness and stored at −20 °C until ready for use.

Extracts were named thusly, for example, DScd is the cd extract of DS, SSet is the et fraction of SS, TTea is the ea fraction of TT, and so on.

### 3.3. Cell Proliferation Assay

The cytotoxic effect of NZ surf clam extracts on three cancer cell lines was measured employing the MTT assay. The cells were seeded in a 96-well plate at a concentration of 1 × 10^5^ cells mL^−1^ using the RPMI medium supplemented with 1 % Penicillin-Streptomycin, 1 % l-glutamine and 10 % fetal bovine serum. After incubation in a humidified 37 °C, 5% CO_2_ incubator (Series II Water Jacket supplied by Thermo Scientific) overnight, the cells were treated by NZ surf clam extracts at a concentration range from 25 to 1000 μg/mL. The cells were further incubated for an additional 24, 48, and 72 h independently at 37 °C. After incubation, MTT stock solution was then added to each well and incubated for a further 4 h. The formazan crystals in each well were dissolved in 100 μL of DMSO. The amount of purple formazan was determined by measuring the absorbance at 540 nm.

#### Data Interpretation

Treated cells with absorbance values lower than the control cells indicate a reduction in the rate of cell proliferation. On the other hand, higher absorbance values indicate an increase in cell proliferation.

%cell viability = {(At − Ab)/(Ac − Ab)} × 100(1)

where, At = Absorbance value of test compound; Ab = Absorbance value of blank; Ac = Absorbance value of control; % cell inhibition = 100 − cell viability.

### 3.4. Cell Apoptosis Assay

The apoptotic effect of NZ clam extracts was determined by the Alexa Fluor^®^ 488 Annexin V staining method and measured by flow cytometer. Cells were seeded onto 6-well plates, at a density of 4 × 10^5^ cells per well and allowed to incubate overnight. The cells were then treated with NZ clam extracts for 7 h at 400 and 600 µg. After treatment, the cells were washed with PBS and harvested using trypsin. Careful measures were taken in order to prevent the loss of detached dead or apoptotic cells. The harvested cells were washed twice with cold PBS, then resuspended in 1 × binding buffer. 4 µL of Alexa Fluor^®^ 488 Annexin V and 1 µL of PI (Alexa Fluor^®^ 488 Annexin V/Dead Cell Apoptosis Kit) were added to each 100 µL of cell suspension, with a slight modification to the manufacturer’s protocols. After the incubation period, 400 µL 1× Annexin-binding buffer was added to each sample and analyzed. Fluorescence-activated cell samples were recorded at 10,000 events using a flow cytometer (Beckman Coulter’s MoFloTM XDP).

### 3.5. Cell Cycle Assay

Cell lines were seeded onto 6-well plates, at a density of 3 × 10^5^ cells per well and allowed to incubate overnight to ensure maximum cell attachment. The cells were then treated with NZ surf clam extracts for 72 h. After treatment, the supernatant was collected, cells were washed with PBS, and treated with trypsin. Careful measures were taken in order to prevent the loss of detached dead or apoptotic cells. The harvested cells were washed twice with PBS at 4 °C, and fixed with ice cold 80% ethanol at −20 °C for no longer than 7 days. The cells were then gently centrifuged for 2 min, after which permeabilizing solution was added to each sample, and incubated for 30 min at 37 °C. After permeabilization, PI was added and incubated for a further 5 min. Fluorescence-activated cells in each sample was recorded at 10,000 events using a flow cytometer (Beckman Coulter’s MoFlo^TM^ XDP).

### 3.6. The Apo-ONE^®^ Homogeneous Caspase-3/7 Assay

The Apo-ONE Homogeneous caspase-3/7 reagents were prepared according to the manufacturer’s recommendations. Cells were seeded onto 96-well plates at a density of 5 × 10^3^ cells per well, and allowed to incubate overnight. The cells were treated with NZ clam extracts for 24 h at 400 and 600 µg/mL. After incubation, an equal volume of Apo-ONE caspase-3/7 reagent was added to each well, and incubated while shaking for 1 h at room temperature. The fluorescence of each well was read at 495 ± 10 (excitation) and 520 ± 10 (emission).

### 3.7. Statistical Analysis

MTT and caspase data were collected from duplicate experiments of triplicate samples. Apoptosis and cell cycle assays were carried out twice, in duplicate. Results are presented as mean ± standard error of the mean and *p* < 0.05 was considered statistically significant. The use of t-test, non-parametric comparison, and 1- and 2- way ANOVA applications were employed. Also, post-analysis Dunnett testing was used to identify differences in data from this study. MTT and caspase data were analyzed using Microsoft Excel. Analysis of Flow cytometry data was performed using Kaluza Analysis 1.3 (Beckman Coulter, Miami, FL, USA).

## 4. Conclusions

This is the first study to describe the preparation of NZ surf clam extracts and their anticancer activities. Results show that cell proliferation was time and concentration dependent, decreasing as both variables increased. The results demonstrate that NZ surf clam extracts possess remarkable bioactivity, responsible for the observed in vitro caspase-dependent apoptosis and disruption of cell cycle progression in the four cancer cell lines. These results may provide a basis for the further investigation and development of NZ surf clam extracts in cancer treatment or treatment supplementation.

## 5. Future Research

This study confirms the anticancer potential of NZ surf clam extracts. Additional data are needed to better understand their bioactivity. The functional components in the extracts require further investigation (isolation, identification, and chemical analysis). Furthermore, an in vivo study will answer the question – whether the extracts’ bioactivities still exist and to what extent in the whole organism. Lastly, it is well known that heat may denature certain proteins and/or other metabolites. Therefore, a study on an alternative clam flesh drying method (versus oven-drying in this study) will be of tremendous importance.

## Supplementary Materials

Supplementary materials can be found at https://www.mdpi.com/2227-9059/7/2/25/s1.
