# Supplementary material for: Cytotoxicity of Extracts from New Zealand Surf Clams Against Organ Cancer Cell Lines
Source: Biomedicines. 2019 Mar 30;7(2):25. doi: 10.3390/biomedicines7020025 (PMC6630374; doi:10.3390/biomedicines7020025)
Supplement: Supplementary file 1 [file biomedicines-07-00025-s001.pdf]

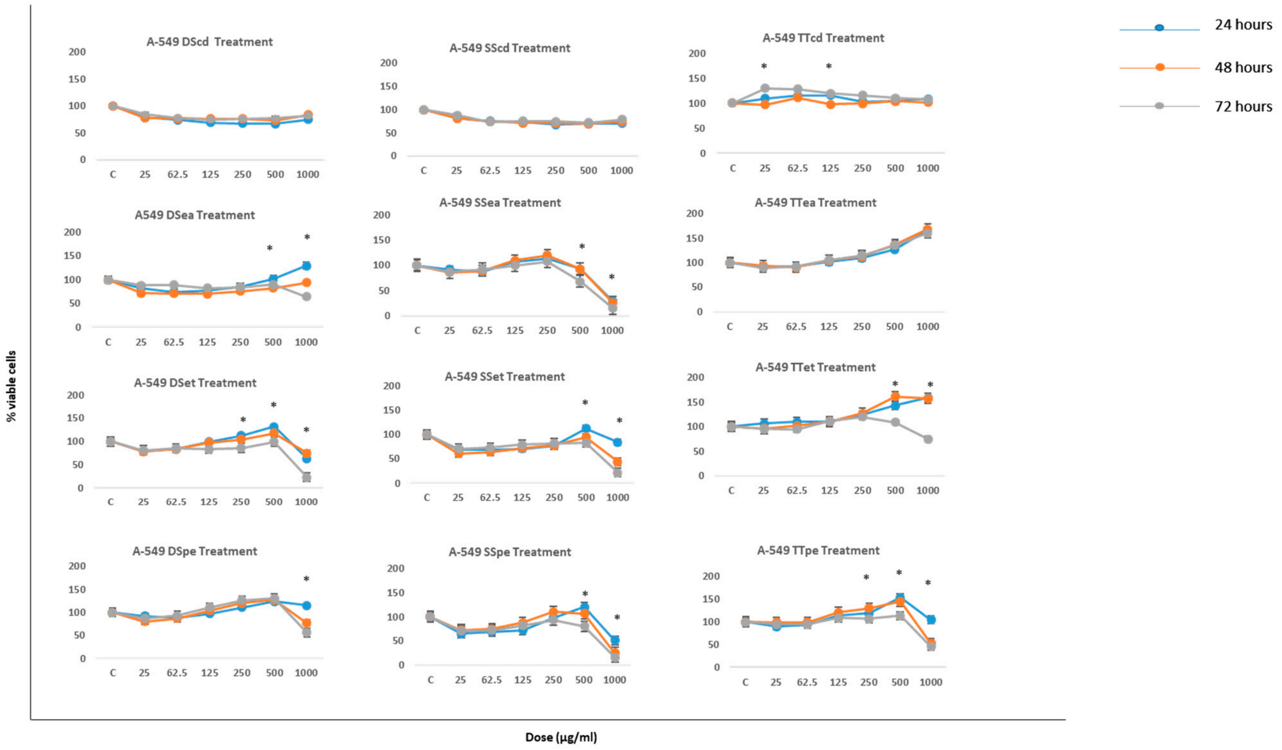

Supplementary Fig. 1a: The inhibitory effect of NZ surf clam extracts on the growth of A549 after an incubation time of 24, 48, and 72 hours. Cells were incubated in the presence of various extract concentrations. A relative cell viability of 100% was designated as the total number of cells that grew after each time point. Each experiment was carried out twice, in triplicates. Data is presented as means ± SD. * indicates statistical significance, $p < 0.05$.

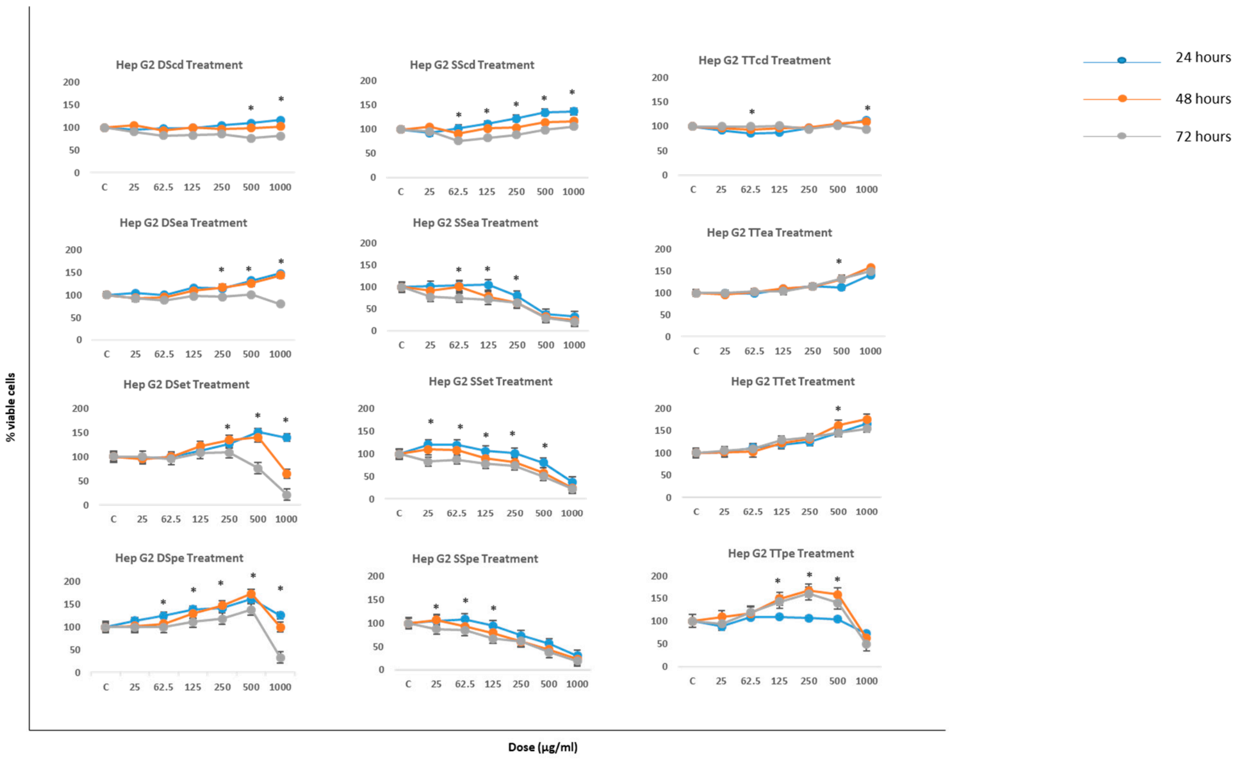

Supplementary Fig. 1b: The inhibitory effect of NZ surf clam extracts on the growth of Hep G2 after an incubation time of 24, 48, and 72 hours. Cells were incubated in the presence of various extract concentrations. A relative cell viability of 100% was designated as the total number of cells that grew after each time point. Each experiment was carried out twice, in triplicates. Data is presented as means ± SD. * indicates statistical significance, $p < 0.05$.

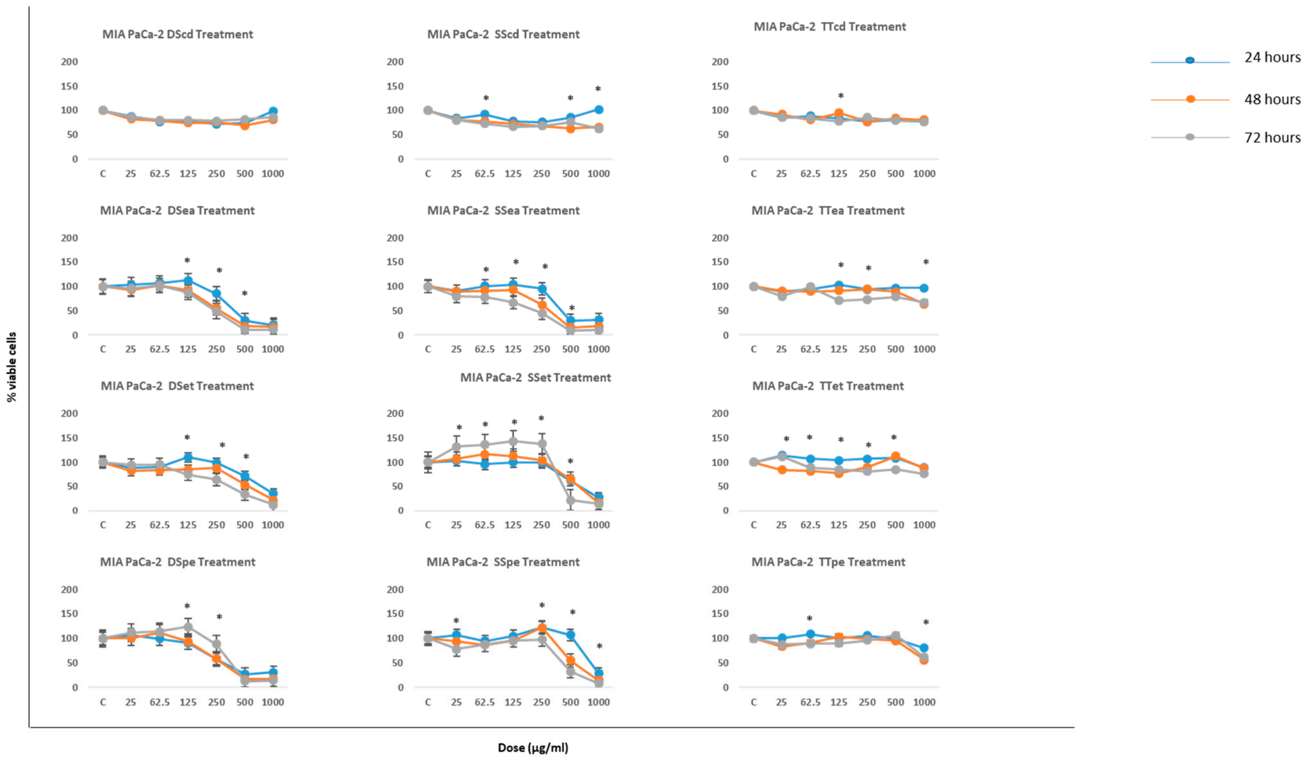

Supplementary Fig. 1c: The inhibitory effect of NZ surf clam extracts on the growth of MIA PaCa-2 after an incubation time of 24, 48, and 72 hours. Cells were incubated in the presence of various extract concentrations. A relative cell viability of 100% was designated as the total number of cells that grew after each time point. Each experiment was carried out twice, in triplicates. Data is presented as means ± SD. * indicates statistical significance, $p < 0.05$.

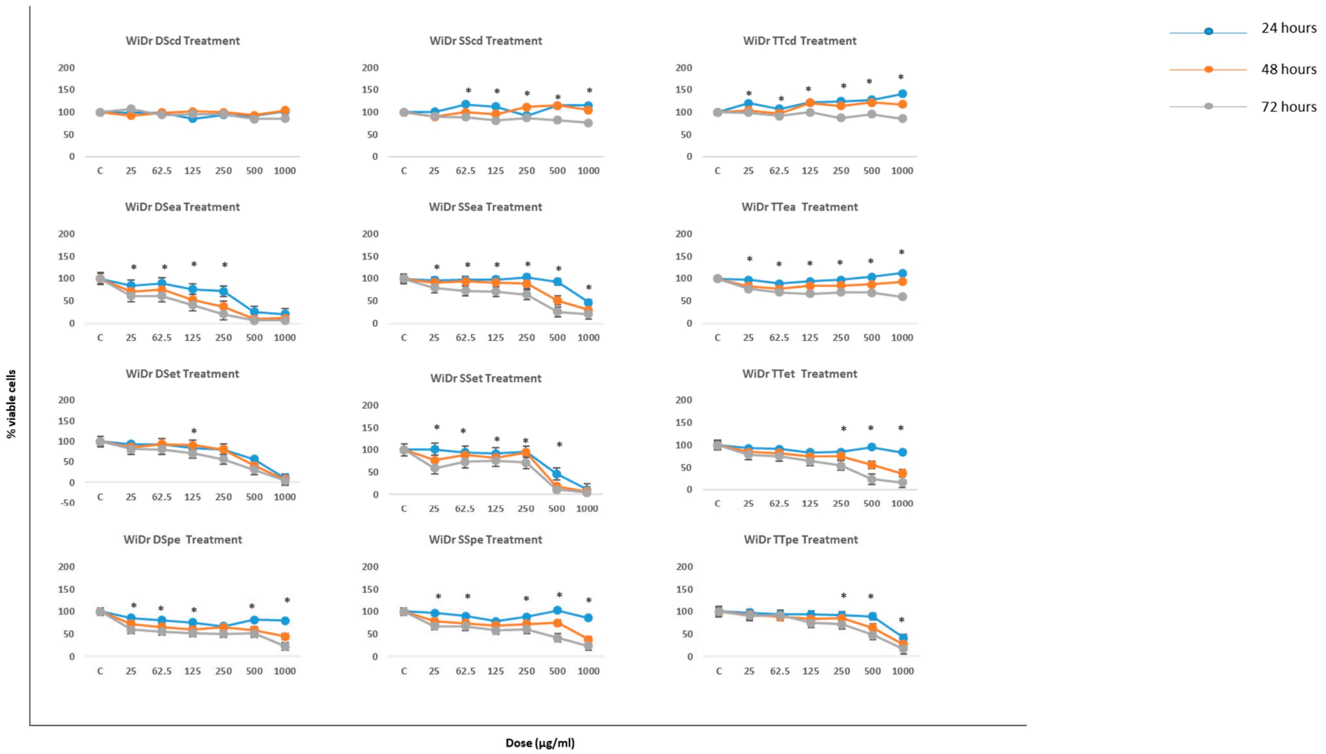

Supplementary Fig. 1d: The inhibitory effect of NZ surf clam extracts on the growth of WiDr after an incubation time of 24, 48, and 72 hours. Cells were incubated in the presence of various extract concentrations. A relative cell viability of 100% was designated as the total number of cells that grew after each time point. Each experiment was carried out twice, in triplicates. Data is presented as means ± SD. * indicates statistical significance, $p < 0.05$.
